# The ethics of shared Covid-19 risks: an epistemological framework for ethical health technology assessment of risk in vaccine supply chain infrastructures

**DOI:** 10.1007/s12553-021-00565-3

**Published:** 2021-06-07

**Authors:** Petar Radanliev, David De Roure, Uchenna Ani, Graca Carvalho

**Affiliations:** 1 Department of Engineering Science, Oxford E-Research Centre, The University of Oxford, 7 Keble Road, OxfordOxford, OX1 3QG UK; 2 Faculty of Engineering Science, STEaPP, University College London, London, England; 3 Centro Algoritmi, Universidade Do Minho, Braga, Portugal

**Keywords:** Covid-19 and healthcare systems, Vaccine supply chains, Ethics of shared risk, Internet-of-things and cyber risk, Ethical supply chain infrastructure, Ethical supply chain design

## Abstract

This article addresses the topic of shared responsibilities in supply chains, with a specific focus on the application of the Internet of Things (IoT) in e-health environments, and Industry 4.0 issues—concerning data security, privacy, reliability and management, data mining and knowledge exchange as well as health prevention. In this article, we critically review methodologies and guidelines that have been proposed to approach these ethical aspects in digital supply chain settings. The emerging framework presents new findings on how digital technologies affect vaccine shared supply chain systems. Through epistemological analysis, the article derives new insights for transparency and accountability of supply chain cyber risk from Internet of Things systems. This research devises a framework for ethical awareness, assessment, transparency and accountability of the emerging cyber risk from integrating IoT technologies on shared Covid-19 healthcare supply chain infrastructure.

## Introduction

The term Internet of Things (IoT) emerged in 1999 [1] and the current adoption of IoT in Covid-19 healthcare services integrates interoperability in technologies such as intelligent healthcare, vaccine and personal protective equipment (PPE) production, manufacturing and supply chains. The Covid-19 has resulted in emergency measures for Covid-19 essential medical products, but these measures resulted in different levels of risk. For example, the emergency measures for authorisation of PPE can be considered as low risk, but authorisation of diagnostic tests can be seen as medium risk. While the emergency use authorisation of oxygen ventilators procurement or installation is high risk and in the case of Covid-19, it resulted in waste of public funds on what are now shown to be unusable medical products’ [2]. This article reviews the ethics of shared Covid-19 risks, from an epistemological perspective for ethical assessment of risk in healthcare technology and vaccine supply chain infrastructures.

Supply chains are defined as the network of organisations involved in the different processes and activities that produce value in the form of products and services delivered to the consumer [3]. IoT-enabled supply chains are defined as a network of digitally connected physical objects that can sense, monitor and interact within and between companies, enabling agility, visibility, tracking and information sharing in the supply chains for timely planning, control and coordination of the supply chain [4].

The motivation for this research emerges from the increased sense that IoT systems generate new types of ethical risks in the supply chain that are not always risk assessed. This prompted an investigation on how the introduction of IoT brings new risks to the security of supply chains. By ethical risks, we refer to the chain of trust in supply chain cybersecurity, e.g., ethical awareness, transparency, and accountability in assessment of supply chain shared risks. In this paper, we expand on the existing definitions of ethical awareness, transparency, and accountability with epistemological analysis. Risk assessment is already a well-defined process, and broadly involves (1) risk analysis – identifying and analysing future negative events; and (2) risk evaluation – predicting the tolerability of such negative events, as subsets of risk management [5]. Although risk assessment is a well-studied process, current risk assessment approaches are inadequate for IoT [6], because of the periodic assumptions that systems will stay the same, and in IoT systems with changing boundaries, high risks can be missed or mistaken. The risk from IoT technology is also changing how supply chain operations are structured. Hence, risk assessment needs to be revised periodically, triggering a challenge to interpret and define the risks in a harmonised way across the supply chain with different  and stakeholders (each with its own methodology).

The framework can be applied to assess cyber risk from third parties operating in a supply chain and to assess cyber risk from IoT devices integrated by third parties in digital supply chains.

## Literature review on ethical assessment of IoT cyber risk

There is an increasing literature on the topic of IoT security [7], [8], but there are very few studies that contribute to the ethics of cyber risks in the IoT-enabled supply chains [9], and the data democracy in supply chains [10]. By ethics of cyber risks, we refer to ethics related to networked IoT systems and what such coupled systems are programmed to do, and how this affects IoT-enabled supply chains. On the other hand, the balance of security and usability, considerations on the necessity of collecting, storing, transmitting data, or shared responsibilities are considered in this paper as questions that define a broader understanding of cyber-ethics.

While the cyber-ethics pertaining to cyber risk from computers, user behaviour and computer programs is regulated by governments and regulatory organisation, the ethics of IoT risk as a topic is not well understood [12], but very relevant as healthcare professionals and patients, along with major medical organisations are adopting advanced IoT solutions to improve operations technology in shared vaccine supply chains. IoT solutions are bound to alter the vaccine supply chain system’s attack surface and introduce new threats which, undoubtedly, have to be considered when developing robust supply chain solutions [12]. But as the IoT is introduced into the supply chain (which has a shared infrastructure), it leads to new ethical issues, because the IoT-enabled supply chains expose new types of cyber risk [13] in the shared healthcare infrastructure. Hence, the integration of IoT into a vaccine supply chain requires ethical reference architectures for managing complexities [14]. Currently however, the existing digital architectures [15–17] lack clarification or guidance [11] on how to approach ethical concerns in the strategic, functional and operational challenges emerging from IoT technologies. This lack of emphasis may also highlight a shared risk where more than one entity is exposed to or can influence the risk. Supply chains and the distributed ownership of IoT devices represent a good example of shared risk in information platforms, connecting medical technology and informatics with the needs of Covid-19 healthcare.

Related literature reports on aspects of digital entrepreneurial ecosystems [18], or digital transformation [19]. Existing literature also addresses the obstacles in technical and management perceptions of enterprise information systems [20], and the business–IT fit in e-procurement systems [21]. However, a quarter of SME’s in the UK do not even possess basic digital skills [22]. The digital problems SMEs face are mainly caused by the barriers imposed to adoption of smart manufacturing technologies, e.g., cost of computing power, cost of implementation or analysis software [23]. Such barriers trigger ethical concerns related to who is responsible for cyber risks in shared systems, such as supply chains. This is an example of what we mean and how we define ethical concerns related to cyber risk in supply chains.

In this article, the ethical assessment of shared cyber risk in IoT-enabled supply chains is conducted through an epistemological framework. The rationale for deploying IoT systems in vaccine supply chains are the opportunities for improved security, efficacy, and low cost in comparison to the risks. Smart manufacturing would create large savings and enable economies of scale [24], presenting new opportunities for fast vaccine production and supply. Smart technologies enable meeting individual patients requirements and create value opportunities, increasing resource productivity, and providing flexibility in healthcare processes. Our understanding of the ethical interaction between humans and IoT [25], is built upon our understanding of cyber-physical systems and human interactions with social machines. The impact of information technology on performance has been related to a flexible production function approach [26]. In addition, the digital product innovation has been investigated within classes of innovation networks [27]. During Covid-19, the vaccine supply chain models have embraced the opportunities from IoT technologies. Real-time enabled IoT systems are already present in vaccine supply chains. However, the ethical concerns regarding the analysis and treatment of risks in an IoT-enabled, shared infrastructure remains to be determined.

The ethical concerns linked to shared risk are amplified by the constantly changing cyber risks in the IoT environment, and the differences in the range of estimated losses possible [28–30]. The concern is that many supply chain participants simply lack an understanding about IoT security threats. Even if all supply chain participants understand IoT risks, there is still an inconsistency in measuring cyber risk [31]; here we view cyber risk as a part of the larger IoT risks. Literature mostly calculates the impact on organisations stand-alone risk, ignoring the cascading impacts of shared infrastructures. Shared risk in infrastructure [32], accompanied with the risk of data pollution [33], and the ‘out of date’ data [34], trigger ethical concerns about machines and products storing information in the network [35]. This generates ethical concerns from who can get access to this information.

Existing literature is dominated by a separation between supply chain models and the emergence of IoT technologies. Very little research has been conducted on the topic of shared risk from IoT systems in supply chains. The epistemological framework in this article is developed to organise existing supply chain models and derives with insights on adapting ethical assessment of shared cyber risk from IoT technologies in supply chains. Existing digital architectures lack clarification on shared cyber risk in individual levels of the strategic, functional and operational supply chain IoT technologies. IoT technologies on the other hand are focusing more on the technical capabilities, disjointed from ethical considerations of shared environments.

The IoT enables the real-time feedback from different users and markets. Unfortunately, this technology comes with cyber risks that can easily be transferred from one supply chain user to another. Hence, such technology requires strong security mechanisms, some of which can be guided by the ethical concerns facing the collaborating organisations. In addition, access control is required for granting or denying requests for information and processing services. Life cycle process is needed for updating the list of assets that are added to the network across multiple timescales. Digital supply chains should also counteract components that have been inappropriately modified to cause a disruption. This process requires a detailed ethical assessment on risk transfer and level of risk acceptance, however ethical assessment of such systems is complex. The reason for this is that digital cyber supply chain networks need to enable awareness, transparency, accountability and assessment of shared risks, constituting the entire system at runtime. Therefore, the digital supply chains need to encompass the ethical assessment in the security and privacy assessment.

The focus of this research is the ethical integration of IoT technologies in shared supply chains. This is inevitable, because IoT creates resource savings, create value opportunities, provide flexibility for the health work force (e.g., remote patient data screening by physicians, nurses, medical physicists, clinical engineers, biomedical engineers). Unfortunately, very little ethical research (if any) has been conducted on the topic of the awareness, transparency, accountability and assessment of IoT cyber risk. Such literature rather represents a juxtaposition of models and studies on IoT supply chain technologies. This paper investigates this juxtaposition by building an epistemological framework, and categorising literature to synthesise knowledge from existing models and studies. Note that in this research, we use the term ‘epistemological framework’ from an ethical point of view, to promote the reassignment of knowledge and beliefs of operations from its traditional place in the domain of knowledge to a new position in the domain of opinion and probability [36]. The framework derives insights into the shortcomings of the methods present in industry and literature, and relates the findings to the topic of awareness, transparency, accountability and assessment of shared cyber risk from IoT-enabled supply chains. Literature review with epistemological reflexivity on supply chain models.

In the literature reviewed, the ethics of cyber risk is often discussed outside of IoT-enabled supply chains. Even a generic supply chain is frequently investigated as a purely event-oriented concept and the consequences of an event are analysed either on a company level or on a supply chain level that creates a cascading effect on the entire network [37]. In this article, we define a supply chain as the entire network involved in the creation and sale of a product, including the delivery to the end user. Some examples of IoT-enabled supply chains include Bluetooth Low Energy beacons that provide information on identity, location and other data, such as temperature and humidity, to a series of collaborating, manufacturing organisations. IoT technologies in supply chains enable operations such as vehicle routing in humanitarian logistics, or quality controlled logistics for perishable products, rerouting products depending on their quality [4]. Ethical discussions in IoT-enabled supply chains usually have referred to ethical sourcing, transparency, and IoT technologies capturing supply chain data, among many other uses. This article however is looking at the ethics of cyber risk in IoT-enabled supply chains. This refers to the risk of IoT devices being compromised in one part of the supply chain network, that affect a different part of the network. For example, a compromise occurs in one organisation that is involved in the creation of a product, and the compromise affects the organisation that is involved in the sale of the product. This specific example can be considered as a security concern, or even a legal concern (e.g., if you have questions of liability). But the example also triggers ethical concerns related to who is responsible for cyber risks in shared systems, such as supply chains. From a technical point of view, the review does not address all the related areas of supply chains, because that would represent too many topics and lead to a lack of focus. Instead, the epistemological review is focused on the ethical best practices, design principles and common approaches. The focus is primarily on identifying concepts related to the ethics in supply chains in relation to cyber risk from IoT technologies. To achieve this, epistemological reflexivity is used to analyse the risk and ethics as a shared cause and the effect, affecting one another in a relationship in which neither can be assigned as causes or effects [38]. Epistemological reflexivity in this article can be defined as continuously challenging the methodological decision and the decisions regarding the findings. Hence, ethics in the review is pursued in different elements of supply chain strategies.

## Epistemological framework for ethical assessment of shared IoT risk in a supply chain infrastructure

In this section, we engage in two activities. First, we describe a framework for ethical assessment of shared IoT risk in supply chains. Next, we investigate the relationships between existing models for ethical design in an IoT environment, and models for ethical supply chain design. Existing models identify the challenges for ethical design in the IoT and ethical assessment of supply chain design, however existing ethical frameworks are not developed to include the assessment of shared risk from IoT. Shared risks require ethical assessment of the current and future impacts of such risk, emerging from IoT products and supply chain designed and/or managed processes. This section focuses on establishment of a clear framework for this task. We concentrate on clarifying the conceptual foundation of the epistemological framework; this enables the study to derive a consistent and solid framework from the extant literature. The process is presented with graphical analysis through which other cases can be studied.

### Definitions of ethical awareness, transparency, accountability in assessment of shared risks

In the proposed framework, the design is considered “ethical” because it enables awareness, transparency, accountability and assessment of shared risks. “Ethical awareness” in this paper is defined as the ability to critically analyse, evaluate, and change our understanding of value and the effects of individual actions to supply chain participants, which also requires transparency. Current definitions of “ethical transparency” are based on *‘ethical implications of the technology used in implementing information transparency’* [39]. Since supply chain is a system, with individual operations structured towards a common goal, the “ethical transparency” in this paper is defined as the ability of individual supply chain participants to crucially assess against its own values, the actions of other participants, in an upfront, visible and honest way. Transparency of actions requires some form of accountability, because without accountability, actions could be presented deceivingly. “Ethical accountability” is a sub-aspect of governance, and a part of the liability of accounting of actions, ethical accountability in this paper is defined as the accountability of one supply chain participant, to inform other participants of actions or decisions, even when such actions would trigger some form of negative event e.g., punishment, penalty. Similarly, to our definition on transparency, our definition on “ethical accountability” is also based on the idea of emerging ethical technologies and project accountability.

### Ethical assessment of supply chain shared risk

The ethical design dictates that strategic supply chain integration requires: consensus on objectives [40–42]; identification of the best level of integration [43]; confirming organisational compatibility [3], willingness to integrate operations [44–47]; and focus on improved collective performance. The focus is already on supply chain integration [43], [44], but ethical complexities remain in prioritising collective as opposed to individual performance improvement. This creates ethical concerns that result in integration obstacles and should be addressed as a priority and ethical strategies should follow the supply chain collective factors but such processes commonly apply limited measurements [48].

**Fig. 1:**
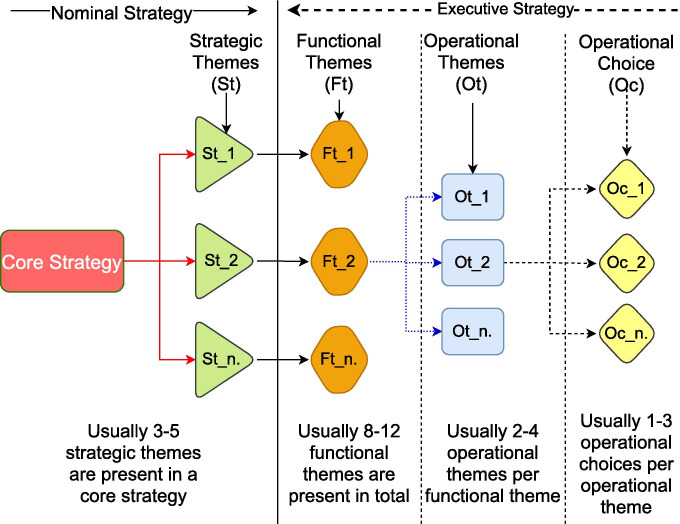
The emerging themes of the epistemological framework for awareness, transparency, accountability and ethical assessment of shared risks in supply chains, originate from analysis and adaptation of existing literature—based on [56]

Epistemological design would enable an understanding on how different types of integration, create different effects [49]. Our epistemological design for an understanding that enables awareness, transparency, accountability and assessment of shared risks is represented in Fig. 1. The framework design builds on the notion that supply chain is a dynamic concept [50] and ethical interdependencies are related in an individual context [51, 52]. The themes emerge from combining findings from different literature stating that the supply chain structural elements are based on a business model [53] as multi-level strategic themes, representing a downwards structured system [54]. Thus, a hierarchical method is applied for shared network design [55], because literature confirms that flow emerges from the strategy, then downwards in the deconstructing of supply chains [42]. This approach was combined with a decomposition approach to create ethical design decompositions [54]. In the deconstructing of design decompositions, following the recommendations from previous research on this topic, the vaccine supply chain strategies are separated between the nominal and executed strategy. In Fig. 1 we describe the process and the ranges that emerged from a detailed ethical review of the designated and actual activities. In this section, synthesised knowledge from the reviewed models is categorised with epistemological reflexivity to derive initial design of our epistemological framework. The themes in Fig. 1 represent the categorisation process of the framework.

The epistemological framework in Fig. 1 differentiates from previous models as it enables investigating awareness, transparency, accountability and ethical assessment of shared risks through the operational activities [57], and not through the designated strategies. The framework represents a generic design presenting the structure for the required assessment of ethical concerns. The scaffolding enables the design process to populate ethical categories and themes with cyber activities, and to compare these activities in an ethical way across the entire supply chain. Exemplar of relevant themes and choices across all the 3 levels is included latter on in Sect. 5. The exemplar describes how the framework in Fig. 1 tackles the issue on ethics of IoT shared risk in supply chains.

**Fig. 2:**
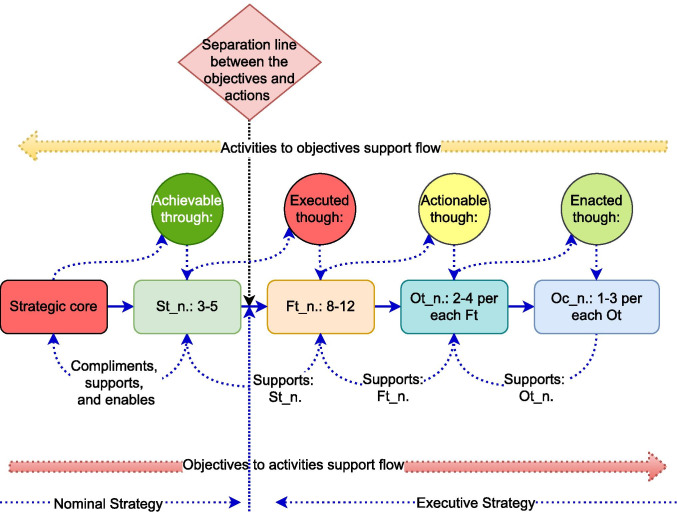
Epistemological framework—integration design that incorporates ethical assessment of shared cyber risks

Prior to populating the structure, the ethical assessment needs to consider how these categories are related and how the integration of these concepts, will affect the ethical assessment of the generic cyber risks. This required advancing the review with models on supply chain integration. Supply chain integration represent a multi-structural decentralised system with active independent elements [58]. The complexities of integrating the themes, can be analysed by applying engineering systems principles [59]. Hence, the integration of the ethical assessment categories in this review applies qualitative research methodologies in combination with engineering design techniques [60]. More specifically, our integration approach follows the recommendation for presenting graphical analysis [61] and is graphically presented in Fig. 2. Similar to the design of Fig. 1, the synthesised knowledge from the reviewed models is categorised with epistemological reflexivity to present the design for the required integration of ethical assessment for shared cyber risks. At this stage of the framework design, the goal was to build the composition of a process that enables the integration of awareness, transparency, accountability in the assessment of supply chain shared risks.

The integration design in Fig. 2, consists of decision that affects the fit between capabilities and strategic objectives, in any given supply chain. Therefore, the epistemological framework can be applied to assess the ethical fit between activities and ethical objectives on shared risk. Such a generic design enables the process of extracting and converting the tacit risks, into explicit risks with a design decomposition process. This design decomposition enables the cyber risk visibility through uncovering of the activities, and not only the factors driving the activities. The design decomposition enables similar and distinct risk activities to be identified, along with the factors driving the technological expansions in supply chain design [50].

## Applying the epistemological framework for assessment of ethical concerns in practice—case study

The findings from the review are applied with a case study research for evaluation of the emerging criteria and outcomes from applying the epistemological framework. The documented process in this section presents the outcomes of the evaluation process.

Case study research was used to populate the scaffolding of the epistemological framework (from Fig. 1 and Fig. 2) and to address the ethical obstacles identified in the literature. The case study research is performed through engagement with participants from Cisco Systems in the USA and Fujitsu in the UK. Cisco and Fujitsu operate as IoT product and services providers for a diverse set of industries. The participating centres depend on multiple participants working as a continuum in the supply chain, which is representative of the diverse ethical issues identified in the review.

## Discussion and main findings

This article presents an update on ethical design in the IoT, focused on ethical design in the IoT, but didn’t discuss the impact of shared risk from complex and coupled IoT systems. We advanced the ethical design of IoT, with an epistemological framework for ethical awareness, transparency, and accountability included in the assessment of shared risk, with a specific focus on supply chain design.

This study argues supply chains must be articulated with ethical consideration of the cyber risks, and with full understanding of the operational and digital capabilities of individual supply chain participant, prior to integration of new IoT technologies.

The epistemological framework addresses this knowledge gap, by integrating ethical awareness, transparency, accountability in supply chain design, and includes ethics in the assessment of shared risks. Since multiple parties are involved in the supply chain, the decision to integrate in IoT technologies must be perceived as joint decision, with ethical assessment of the cyber risks, and include all supply chain parties.

## Conclusion

At a higher analytical level, this article focused on developing a framework to provide guidance for governments, medical organisations and healthcare practitioners on how the introduction of IoT brings risks to the security of vaccine supply chains. The verification of the epistemological framework is conducted through interviews and workshops with experts from Cisco and Fujitsu in the field of supply chain and IoT technology. The industrial case study is also informed by the sustained engagement with a broad set of user partners for a wide range of private sectors, government agencies, and charities at international scale.

### Limitations and further research

The paper focuses on introducing the well-known generic supply chain processes, while considering the peculiarities of Covid-19 vaccine production and supply chains, and the IoT ecosystem in the pandemic management design process. The design is informed and verified through interviews, an approach that needs to be further examined and validated with regards to the results the epistemological framework provides.

## Data Availability

The data used in include in the article.

## Code availability

No code was used in this article.
